# Virus Cooperation, ZIKV Viremia and in Utero Fetus Infection

**Published:** 2019-08-22

**Authors:** Pascal J. Goldschmidt-Clermont, Jean-Loup Romet Lemonne, Arnaud Fontanet, Mario Stevenson

**Affiliations:** 1 Alzady International LLC, Dean Emeritus, Professor of Medicine Emeritus, Miller School of Medicine University of Miami, Miami, Florida 33136.; 2 Global Development, Institut Pasteur, 708 Greenwich street #4C, New York, NY 10014.; 3 Institut Pasteur, Emerging Diseases Epidemiology Unit, Paris, France.; 4 Conservatoire National des Arts et Métiers, Paris, France.; 5 Department of Medicine, Infectious Diseases Division, and HIV/AIDS and Emerging Infections Institute, Miller School of Medicine, University of Miami, Miami, Florida 33136.

**Keywords:** virus cooperation, zika virus, pregnancy, placental, fetus infection, women and child health

## Abstract

With growth and exponential globe trotter traveling of the human population, and many more conducive factors, the likelihood of merging and melting viruses capable of infecting humans in a cooperative fashion, has increased markedly. Hence, viruses that were limited to a particular region of the planet, or to certain population groups, have become capable of infecting humans on a pandemic scale. Some viruses not only can infect pregnant women, but also expand to the amnotic fluid and fetus. With this review, we will reflect upon some examples of known viral cooperations, as well as new ones that have the potential for compromising human health and survival of the fetus in utero.

## Introduction to Virus Cooperation

**Figure 1:**
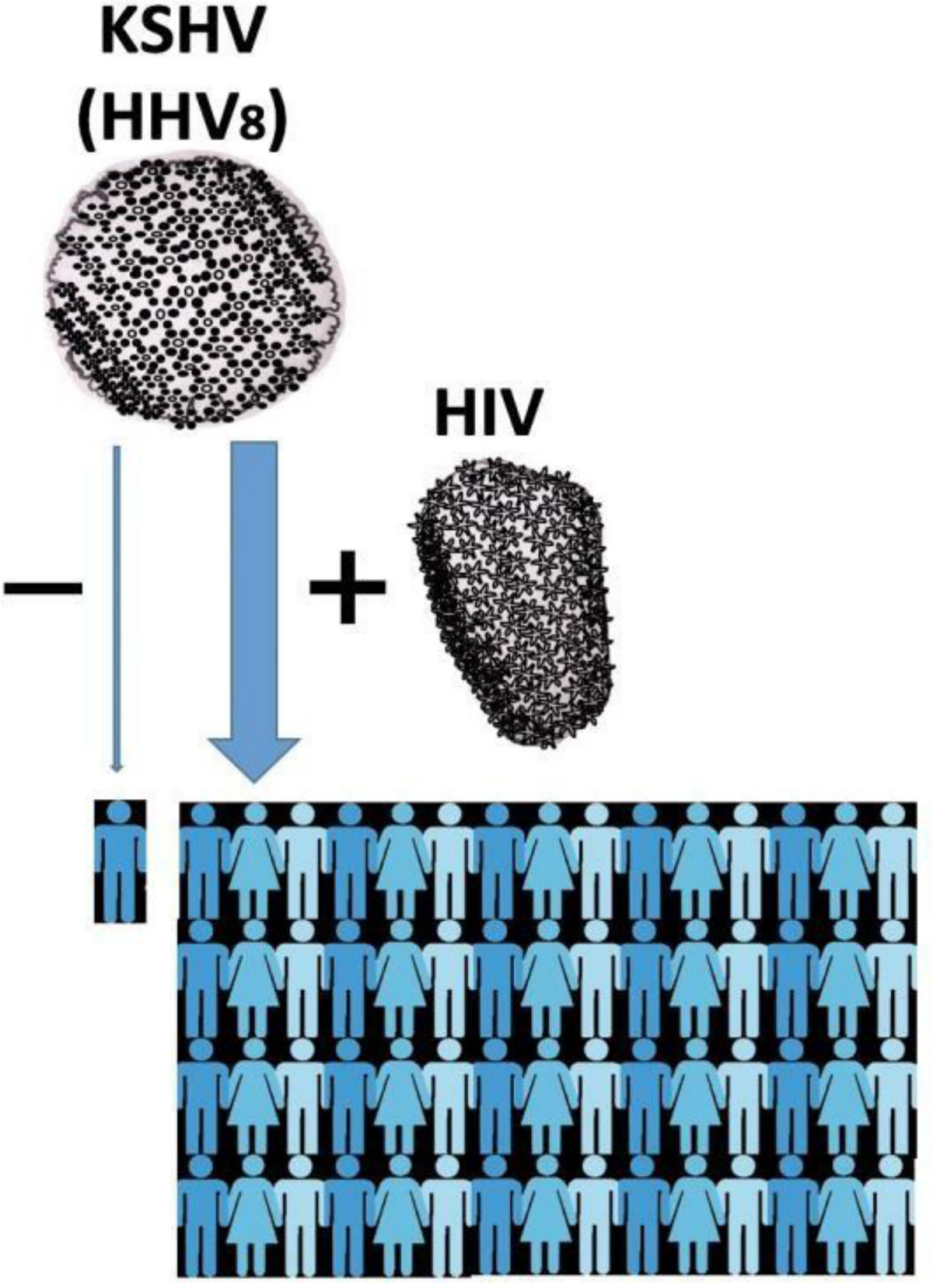
HIV and KSHV cooperation. As the pandemic of HIV was spreading worldwide during the last quarter of the 20^th^ century, Kaposi Sarcoma caused by KSHV (HHV8) has expanded from an infrequent illness for elderly men in the Mediterranean Basin and Africa, to a ubiquitous cancer affecting millions of men and also women, and still is the leading cancer for AIDS patients in sub-Saharan Africa, as a result of “virus cooperation” between KSHV and HIV.

Parnassus Avenue, a typical summer morning of 1983, the UCSF dermatology clinic is opening, there is a line of mostly young men, many of whom display one or multiple discolored (purplish) lesions of their skin or mucosa, known as Kaposi’s sarcoma (1). It was before the AIDS and Kaposi’s viruses were discovered, and for patients newly diagnosed with AIDS, time from AIDS diagnosis to death was then 12 months (2). The virus that triggers the Kaposi’s lesions turned out to be of the Herpes family, Human Herpes Virus 8 (HHV8), better known today as Kaposi’s Sarcoma-associated Herpes Virus (KSHV) (3). Before the AIDS pandemic, Kaposi’s sarcoma was a rare disorder, found on the skin of mostly elderly men in countries surrounding the Mediterranean Sea, Middle-Eastern Europeans, and in Africa (3). Since the beginning of the AIDS pandemic, millions of patients have suffered from Kaposi’s sarcoma, women and men almost equally. Although it has become rare again in countries where AIDS treatment is readily available and applied effectively (4), it remains the most frequent AIDS-associated cancer in sub-Saharan Africa (Figure1).

Why would two viruses like HIV-1 and KSHV expand concurrently within the human population? Both viruses can be transmitted through sexual contact, so they may naturally be more commonly found among individuals with at-risk sexual behaviors. However, there may be more to this cooperation than that, with evidence in experimental models that the replication of one virus may enhance the replication of the other, and that there is loss of T-cell proliferative response (a critical step of immune reaction) to KHSV in HIV-infected men (5).

Another virus that has expanded markedly through parenteral transmission within the human population during the past fifty years is the hepatitis C virus (HCV), with multiple cases of co-infection with HIV among injecting drug users. In this case, HCV-associated complications, and particularly hepatic fibrosis, are accelerated during co-infection with HIV, whereas the impact of HCV on HIV infection is less clear, if any (6–8). While in HAART-treated AIDS patients, Kaposi’s sarcoma has nearly vanished, there is only modest improvement of hepatitis C clinical progression as a result of HIV therapy, and HAART is often hepatotoxic. Hence, the net benefit from HAART on the liver condition of HCV and HIV infected patients remains controversial (6–8). Perhaps, an even more dramatic example of cooperation between viruses is the case of hepatitis delta virus (HDV), which can become infectious to humans only in the presence of hepatitis B virus infection (9).

These examples illustrate a major challenge for 21st century medicine: the cooperation between viruses. Most of the viruses that now afflict the population predated humans and have infected other animals prior to infecting humans. But what has changed drastically in recent years with the growth of the human population, the connectivity of our world, and the substantial increase in the number of world travelers, is the probability for previously distant viruses to interact, and thus possibly cooperate, to infect humans.

## Virus Cooperation and Pregnancy

A recent “emerging infection” issue that infectious disease specialists, scientists, obstetricians and pediatricians alike are struggling with and puzzled by, is how and why does the Zika Virus (ZIKV) demonstrate such an unusual proclivity for the fetal human brain, with grave consequences for the fetus such as microcephaly and other major brain abnormalities (10–13). In a study performed in Rio de Janeiro, Brazil, amongst pregnant women that had symptomatic, confirmed, ZIKV infection, 42% of life offspring were found to have grossly abnormal clinical features, brain imaging, or both, including 4 with microcephaly (14). Microcephaly is the most obvious of the birth defects reported in ZIKV-infected newborns, and in Brazil, especially in the Province of Pernambuco (North-East of Brazil, on the Atlantic Ocean), cases of microcephaly have increased by 2,300% between 2014 and 2015 (15). Microcephaly and other neurological defects of the fetus and newborn are more commonly associated with infections taking place during the first or early second trimester of the pregnancy (10), but can apparently happen during all three trimesters (14). Other clinical symptoms include ocular lesions, congenital contractures, and hypertonia with extra-pyramidal manifestations (11).

When infected with ZIKV^BR^, predisposed Swiss Jim Lambert mice (SJL/J, with a splice-site mutation in the Dysferlin gene), but not wild type mice, were able to reproduce some ocular and cortical malformations reminiscent of the human fetal abnormalities seen in ZIKV infected fetus, including reduced numbers of brain cells, brain cells that often displayed a “vacuolar nuclei” appearance (consistent with accelerated cell apoptosis/necrosis), and reduced cortical layer thickness (16).

In another model, where ZIKV was injected intra-vaginally in pregnant mice, replication in the vaginal mucosa was observed, with evidence of intrauterine growth restriction and fetal brain infection (17). Furthermore, when IFnar1−/− mice were used, the extent of virus replication in the vaginal mucosa was much stronger, viremia and placental infection ensued, and severe intrauterine fetal growth limitation and brain infection resulted, with markedly increased fetal death (17). This model suggests that ZIKV replication in the proximity of the uterine cavity could result in more frequent placental infection and brain abnormalities in the fetus. Sexual transmission of ZIKV has been documented in Humans (10,12–14), although its impact on the fetus and role in microcephaly and other brain abnormalities will need to be established.

Pregnant non-human primates (Macaca mulatta) infected with a ZIKV isolate demonstrate prolonged maternal viremia (18). This isolate was from Rio de Janeiro where it had been associated with high rate of fetal brain malformation, and was injected to macaques at relatively low challenge dose of a minimally passaged virus stock (18). ZIKV could be found in the blood of pregnant macaques beyond day 7 post infection, at a time when the blood of non-pregnant macaques had become sterile after similar exposure to ZIKV. Amniotic fluid infection was also detected in nearly 50% of dams, and in utero fetal deaths were observed (18), thus reproducing many features of the ZIKV infection of pregnant women.

**Figure 2:**
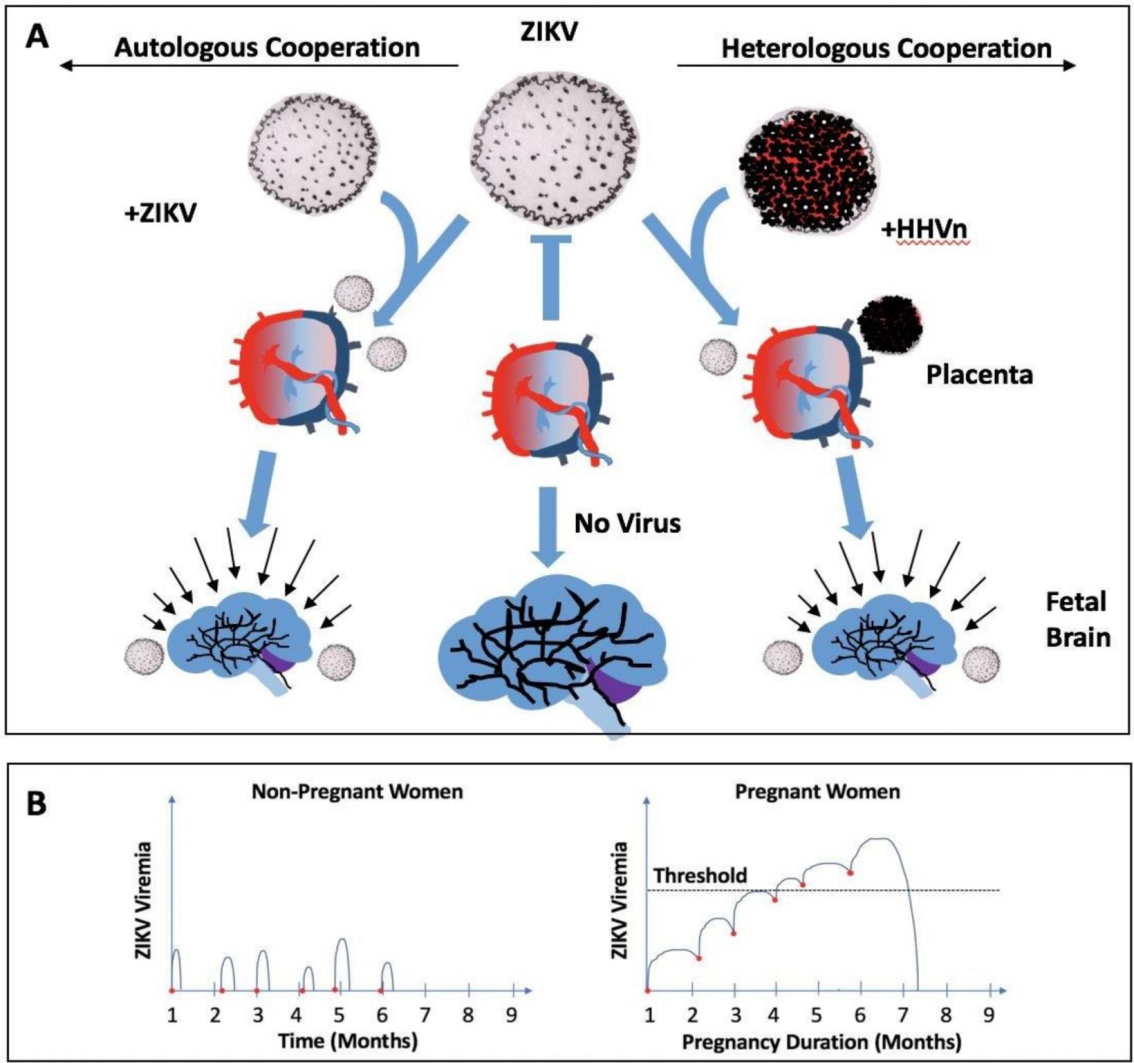
Virus cooperation hypothesis for ZIKV infection of the placenta and the fetus brain. A. The prolonged viremia of ZIKV seen in non-human primates (18) might allow for accumulation of the virus load resulting from consecutive exposures to bites from Ae. Aegypti mosquito (carrier of ZIKV). The resulting viremia overwhelms the placental defense against the virus (autologous cooperation). Alternatively, a prior or concurrent infection (reactivation) of a pregnant woman with a Human Herpes Virus (HHVn) capable of infecting the placenta, might weaken placental resistance to ZIKV, thus creating an opportunity for ZIKV to invade placenta and fetus even in the presence of lower ZIKV viremia (heterologous cooperation). In both cases, ZIKV ends up infecting the brain (the proclivity of ZIKV for the brain of the unborn fetus results in microcephaly as shown), and other fetal tissues. In the absence of such viral cooperation, the placenta is readily capable of blocking ZIKV and thus protecting the fetus1. B. Theoretical graph showing successive mosquito bites (red dots) in a non-pregnant woman (left panel) resulting in limited viremia, versus successive bites in a pregnant woman (right panel), showing the impact on viremia. The challenge created by pregnancy to eliminate ZIKV from the mother’s blood results in a cumulative viremia that exceeds the threshold (dotted line) for infection of the placenta and fetus. ^1^ Artist rendering of virus capsids for figures 1 and 2 were inspired by the image reconstruction of the viral capsids as defined by electron cryomicroscopy work: (a) KSHV: Trus BL, Heymann B, Nealon K, Cheng N, Newcomb WW, Brown JC, Kedes DH, Steven AC. Capsid structure of Kaposi’s sarcoma-associated herpersvirus, a gammaherpesvirus, compared to those of an alphaherpesvirus, herpes simplex virus type 1, and a betaherpesvirus, cytomegalovirus. J Virology 75:2879–2890, 2001. (b) HIV: Zhao G, Perilla JR, Yufenyuy EL, Meng X, Chen B, Ning J, Ahn J, Gronenborn AM, Schulten K, Aiken C, Zhang P. Mature HIV-1 capsid structure by cryo-electron microscopy and all-atom molecular dynamics. Nature 497:643–646, 2013. (c) ZIKV: Sirohi D, Chen Z, Sun L, Klose T, Pierson TC, Rossmann MG, Kuhn RJ. The 3.8 Å resolution cryo-EM structure of Zika virus. Science 10.1126/science.aaf5316, 2016. (d) HCMV: Butcher SJ, Aitken J, Mitchell J, Gowen B, Dargan DJ. Structure of the human cytomegalovirus B capsid by electron cryomicroscopy and image reconstruction. J Structural Biology 124:70–76, 1998

While the ZIKV epidemic in Latin America will result in tens of thousands of babies with neurological abnormalities (19), it is not clear why only a subgroup of women, infected with ZIKV during pregnancy, show susceptibility for having babies with brain complications. Likewise, it is not clear why the inhabitants of some regions, like the Province of Pernambuco in Brazil, displayed such strong proclivity of the infecting ZIKV for the nervous system of the unborn child. It suggests, perhaps, a role for co-factors, among which co-infection with other viruses could be one contributing factor (Figure 2). Hence, clues from existing epidemic surveys are being researched to establish plausible hypotheses that can be tested with translational and clinical research. One intriguing finding from the Brazilian epidemic was the evidence of prior infection with Dengue, confirmed in nearly 90% of pregnant women (14), and occurred at such high frequency whether the fetus presented anomalies or not. ZIKV is a member of the genus Flavivirus of the Flaviviridae family, classified as an arbovirus (arthropod-born), which includes Dengue (DENV), Japanese Encephalitis (JEV) viruses, West Nile (WNV), Yellow Fever (YFV), and are closely related to, and share symptoms with, the Chikungunya virus (CHICV) which is a member of the Togaviridae family (19–21). Hence, it has been speculated that prior or concurrent infection with another one of these viruses could somehow facilitate placental infection by ZIKV and brain defects. One reported mechanism could involve antibody-dependent enhancement (ADE) of virus infection by ZIKV (21). But so far, no such acute co-infection involving ZIKV and another virus of the arbovirus group seems to account for fetal susceptibility, and for ZIKV proclivity for their nervous system (14).

While the concept of general immune suppression (as in patients post-organ transplant) during pregnancy has been effectively rejected as overly simplistic, (22), the prolonged ZIKV viremia observed in infected pregnant primates (18) could be secondary to a specific situation of immune “tolerance” relative to this particular virus during pregnancy. Such prolonged viremia could be a major factor involved in secondary amniotic fluid and fetus infection (18). In situations where ZIKV-infected mosquito bites are more frequent, the prolongation of viremia in pregnancy may lead to cumulative rise ZIKV viremia over time, to a point of overwhelming the natural defense of the placenta and consequent infection of the fetus brain (autologous cooperation) (Figure 2).

The mechanism for immune tolerance to ZIKV in pregnancy is unknown, but infection of the placenta and fetus could be secondary to virus cooperation, autologous or heterologous cooperation. Indeed, concurrent infections of the human fetal brain with ZIKV and HHV have also been reported (23).

Before the Brazilian ZIKV epidemic, another ubiquitous virus, Cytomegalovirus (CMV, HHV5) (24), which, like KSHV (HHV8) and Epstein Barr (HHV4) virus, is a member of Human Herpes Virus family (Herpesviridae), was well known for infecting the placenta of pregnant women and causing nervous system damage, although not with the prevalence and severity of ZIKV (14,25). Among newly infected women, the rate of transmission *in utero* of CMV is very high (between 30 and 40%), and fetal abnormalities occur mainly with infections that occur during the first trimester (24). Intriguingly, other members of the Herpes Virus family can also infect the human placenta, although less frequently than CMV. Human Herpes Virus 1, 2, 6, Epstein Barr Virus (EBV), and even KSHV, all have been shown, as with CMV, to infect the placenta (23,26).

It is tempting to speculate, and readily testable, that co-infection with ZIKV and one of the Human Herpes Viruses could potentiate the likelihood of placental transmission of ZIKV, and perhaps aggravate consequential infection of the fetal brain. This hypothesis has been tested clinically already for CMV, and results so far do not support cooperation between acute CMV infection and ZIKV, but it has not been tested for other Human Herpes Viruses that are known to infect the placenta (23,26). Furthermore, a prior CMV infection may cause diminished bystander activation of natural killer (NK) cells (27), thereby reducing resistance to other infecting viruses such ZIKV, even in the absence of acute CMV infection.

Upon co-infection of ZIKV with HHV-2 (HSV-2) in mice, HHV-2 enhanced placental sensitivity to ZIKV, and allowed ZIKV to breach the placental barrier and access the fetus (28). If such a hypothesis and animal experiments were to be confirmed in humans, pregnant women infected with ZIKV could be treated with available drugs that control Human Herpes Virus infections. For sure, more work needs to focus on how the placental barrier can be broken so effectively for some pregnant women infected with ZIKV (29).

## Conclusion

Viruses display an exquisite disposition for frequent mutations, which they rely upon to assure their survival in adverse conditions, as well as their ability to adapt to new hosts in the case of zoonotic infections. At times, they even engage in a process of “hypermutation”, whenever surrounding conditions are changing or challenging. For example, when its transmission amongst humans is facilitated, it is known that a virus can become more aggressive and proliferates faster. A recent study on ZIKV has suggested that a single mutation that occurred in 2013 could explain the heightened proclivity of ZIKV for mouse and human brains (30).

Alternatively, if transmission is a challenge, the virus can adopt a more chronic, benign gestalt. Hypermutation may also explain why KSHV became so much more virulent and contagious than it was before the onset of the AIDS epidemic, and why it was able to infect equally men and women, rather than having greater infectivity for men (31,32). Hence, viruses adapt to their environment through phenotypic changes that are the result of genetic mutations. In some cases, genetic changes result in creation of new genes. In order to infect humans, the ancestors of the lentiviruses evolved genes that neutralize antiviral proteins of the host that themselves evolved to oppose threats from exogenous pathogens (33). At the gene level, alterations in viral envelope genes could lead to altered host cell tropism and allow the virus to take up residence in immune-privileged sites such as the CNS.

The human genome does not undergo much mutation, and certainly never to the extent observed in viruses (even within tumor tissues). Without such mutational plasticity and rapid phenotypic adaptation, human resilience and adaptability instead rely on individual and collective inventiveness and creativity (human “hyperideation” as a corollary for viral “hypermutation”) to adapt to environmental challenges, such as antimicrobial drugs/vaccination campaigns used to combat pandemic infections. Human endeavors in science and research are our best solution to adapt to the changing battlefield involving microbes and their cooperation that will continue to impact human health, starting in utero, throughout the 21^st^ century.

## Clinical Implications

For women, ZIKV infection is a dreadful health issue because of the risk of infection of the placenta, the fetus and consequent condition of their children. ZIKV is transmitted to humans mostly through the bite of Aedes aegypti mosquitoes. This mosquito feeds mainly at dusk and dawn, but can bite at any time of the day, hence bed nets at night are not sufficiently protective. For pregnant women (from first trimester to delivery), the safest protection is to stay, or move, away from endemic area, where the chance of getting bitten by Ae. Aegypti carrying ZIKV multiple times is high (Figure 2). If “away” is not an option, then getting rid of stagnant water (especially inside the house, toilets, showers etc.), long sleeve clothing, screens and bed nets, use of insect repellants containing DEET (N, N-diethylmetatoluamide, 20%−30%) or *p*-menthane-3,8-diol (from lemon eucalyptus), all were shown effective in reducing bites of Ae. Aegypti mosquito, and hopefully below the threshold of viremia required for placenta and fetus infection.
